# Treatment of Persistent Postconcussion Syndrome With Repetitive Transcranial Magnetic Stimulation Using Functional Near-Infrared Spectroscopy as a Biomarker of Response: Protocol for a Randomized Controlled Clinical Trial

**DOI:** 10.2196/31308

**Published:** 2022-03-22

**Authors:** Sané du Plessis, Ibukunoluwa K Oni, Andrew P Lapointe, Christina Campbell, Jeff F Dunn, Chantel T Debert

**Affiliations:** 1 Department of Clinical Neurosciences Cumming School of Medicine University of Calgary Calgary, AB Canada; 2 Hotchkiss Brain Institute Calgary, AB Canada; 3 Department of Radiology Cumming School of Medicine University of Calgary Calgary, AB Canada

**Keywords:** concussion, mild traumatic brain injury, persistent postconcussion syndrome, repetitive transcranial magnetic stimulation, functional near-infrared spectroscopy, traumatic brain injury, TBI, brain injury, brain, symptom burden, mental health, quality of life, neuroscience, neurology

## Abstract

**Background:**

Approximately one-third of all concussions lead to persistent postconcussion syndrome (PPCS). Repetitive transcranial magnetic stimulation (rTMS) is a form of noninvasive brain stimulation that has been extensively used to treat refractory major depressive disorder and has a strong potential to be used as a treatment for patients with PPCS. Functional near-infrared spectroscopy (fNIRS) has already been used as a tool to assess patients with PPCS and may provide insight into the pathophysiology of rTMS treatment in patients with PPCS.

**Objective:**

The primary objective of this research is to determine whether rTMS treatment improves symptom burden in patients with PPCS compared to sham treatment using the Rivermead postconcussion symptom questionnaire. The secondary objective is to explore the neuropathophysiological changes that occur following rTMS in participants with PPCS using fNIRS. Exploratory objectives include determining whether rTMS treatment in participants with PPCS will also improve quality of life, anxiety, depressive symptoms, cognition, posttraumatic stress, and function secondary to headaches.

**Methods:**

A total of 44 adults (18-65 years old) with PPCS (>3 months to 5 years) will participate in a double-blind, sham-controlled, concealed allocation, randomized clinical trial. The participants will engage in either a 4-week rTMS treatment protocol or sham rTMS protocol (20 treatments). The left dorsolateral prefrontal cortex will be located through Montreal Neurologic Institute coordinates. The intensity of the rTMS treatment over the left dorsolateral prefrontal cortex will be 120% of resting motor threshold, with a frequency of 10 Hz, 10 trains of 60 pulses per train (total of 600 pulses), and intertrain interval of 45 seconds. Prior to starting the rTMS treatment, participant and injury characteristics, questionnaires (symptom burden, quality of life, depression, anxiety, cognition, and headache), and fNIRS assessment will be collected. Repeat questionnaires and fNIRS will occur immediately after rTMS treatment and at 1 month and 3 months post rTMS. Outcome parameters will be analyzed by a 2-way (treatment × time) mixed analysis of variance.

**Results:**

As of May 6, 2021, 5 participants have been recruited for the study, and 3 have completed the rTMS protocol. The estimated completion date of the trial is May 2022.

**Conclusions:**

This trial will expand our knowledge of how rTMS can be used as a treatment option of PPCS and will explore the neuropathophysiological response of rTMS through fNIRS analysis.

**Trial Registration:**

ClinicalTrials.gov NCT04568369; https://clinicaltrials.gov/ct2/show/NCT04568369

**International Registered Report Identifier (IRRID):**

DERR1-10.2196/31308

## Introduction

Concussion incidence has been rising among Canadians with the Ontario Physician Billing codes reporting an annual incidence of 1.2% of the population [1]. According to the Canadian Community Health Survey, over the last 2 decades, the number of concussions or other brain injury reports among Canadians is increasing, with 1 in 200 Canadians reporting a concussion or other brain injury to be their most disabling injury [2]. While many recover quickly from concussions, approximately one-third of patients with concussion have prolonged symptoms [3]. The symptoms that these individuals experience are collectively known as persistent postconcussion syndrome (PPCS), and can include cognitive, physical, and emotional impairments [4].

Transcranial magnetic stimulation (TMS) is a tool used to provide noninvasive electromagnetic induction through magnetic fields to the brain in conscious humans [5]. In general, single pulse TMS is used to explore brain function while repetitive TMS (rTMS) is used to change brain activity that lasts well beyond stimulation. rTMS has been shown to be an effective treatment for depressive disorders [6] as well as several other neurological conditions [7,8]. Furthermore, a recent systematic review showed that rTMS may be an effective treatment for patients experiencing PPCS, but studies with larger sample sizes are required to further support this evidence [9].

There is potential in using rTMS to improve symptoms for patients with PPCS, but the mechanism behind rTMS treatment for PPCS remains unknown. Recent studies have suggested that rTMS treatment modifies recovery pace and improves symptomatology [10,11]. Currently, very few studies have looked at biomarkers for rTMS treatment response in patients with PPCS. Our group previously conducted a 2-patient case study demonstrating how functional near-infrared spectroscopy (fNIRS) may be useful as a sensitive tool to predict treatment response to rTMS in patients with PPCS [12]. fNIRS is a noninvasive imaging technique that measures the differences in the absorption of local oxygenated and deoxygenated hemoglobin in the brain. The changes in cerebral tissue oxygenation are then used to monitor brain activity [13,14]. fNIRS has demonstrated comparability to functional magnetic resonance imaging for reliably detecting changes in cerebral vascular reactivity [15], and several studies have already examined the use of fNIRS for assessing concussions [15-17] and PPCS in adults [18]. Previous work assessing pediatric and adult patients with PPCS have shown reduced connectivity in both frontal and motor regions when compared to healthy controls during rest as well as during a finger tapping exercise and working memory task [18,19]. These findings are comparable to literature on functional magnetic resonance imaging, which has shown a reduction in connectivity in patients with mild traumatic brain injury during both resting state and task-based measures [20-22]. fNIRS has many advantages to being used in the clinical setting because of its portability and ability to replace other neuroimaging techniques that may be cost prohibitive.

The primary objective for this study is to determine if the application of rTMS treatment to the left dorsolateral prefrontal cortex (DLPFC) in patients with PPCS will significantly improve postconcussion symptoms using the Rivermead postconcussion symptom questionnaire (RPQ) compared to sham treatment. The second objective of the study is to explore the pathophysiology of rTMS response in participants with PPCS using fNIRS immediately following rTMS treatment and at 1-month and 3-month posttreatment follow-ups. An exploratory aim of the study is to determine whether rTMS treatment to the left DLPFC will improve quality of life, anxiety, depressive symptoms, fatigue, posttraumatic stress, and function secondary to headaches compared to sham treatment. We hypothesize that participants who show a clinically significant improvement in PPCS symptoms will also show an improved hemodynamic response in fNIRS recordings.

## Methods

### Study Design

This is a double-blind, sham-controlled, concealed allocation, randomized clinical trial. Recruitment will occur through 3 locations in Calgary, Alberta, Canada including the Calgary Brain Injury Program, the University of Calgary Sports Medicine Centre concussion clinic, and the Calgary Pain Program. The estimated study duration is from March 2021 to May 2022. Participant enrollment, fNIRS, and rTMS treatment will be completed by authors SD and CC.

### Ethics Approval and Trial Registration

This study was approved by the University of Calgary Conjoint Health Research Ethics Board (REB15-1786). All participants will sign informed consent prior to study participation. This study has been registered with ClinicalTrials (NCT04568369).

### Participants

All participants will be between the ages of 18 and 65 years. The participants will meet the inclusion and exclusion criteria explained in this section. Inclusion criteria include a diagnosis of PPCS based on the International Classification of Diseases (ICD)-10 criteria for at least 3 months to a maximum of 5 years [23]. The ICD-10 states that PPCS must be followed by head trauma with loss of consciousness as well as any 3 of the following symptoms: feelings of unwellness (ie, headaches, dizziness, general malaise, and excessive fatigue or noise intolerance), emotional changes (ie, irritability, emotional lability, depression, and anxiety), difficulty concentrating or performing mental tasks or memory complaints, insomnia, reduced tolerance to alcohol, preoccupation with the above symptoms, and fear of permanent brain damage [23]. Current pharmacologic management will be maintained without change during the treatment study (ie, consistent use of triptans, opioids, tricyclic antidepressants, and antiseizure medications). Participants undergoing Botox treatment will undergo rTMS treatment 6-8 weeks following their injection, which is around the time of peak Botox efficacy [24]. Exclusion criteria comprise a history of prior TMS therapy, TMS-related contraindications (pacemaker, metallic implants, and large intracranial lesion), change in medication, and other neurological or mental health medical conditions such as structural brain disease, previous seizure, psychiatric disorders excluding depression and anxiety (eg, schizophrenia and bipolar disorder), liver or kidney disease, malignancy, uncontrolled hypertension or diabetes, and pregnancy.

### Study Procedure

**Figure 1:**
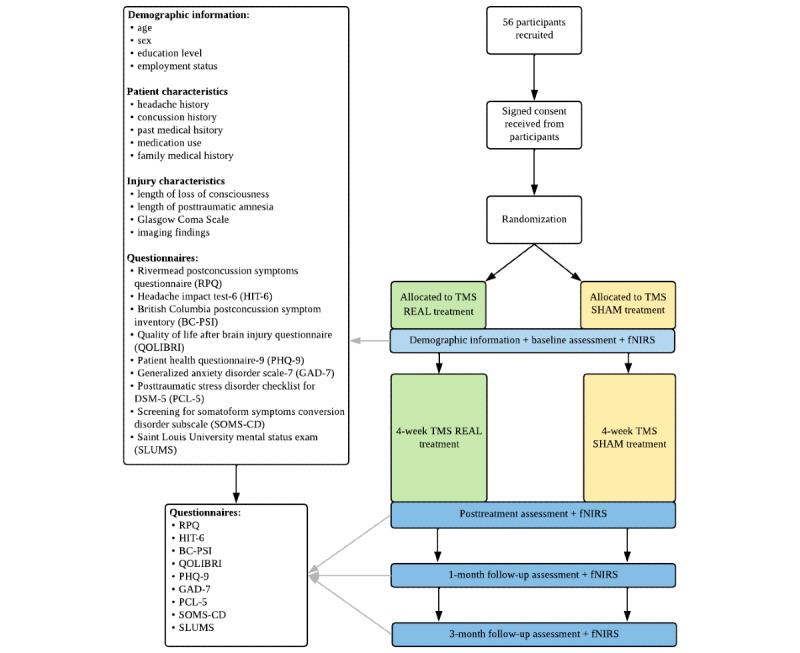
Study design protocol. DSM-5: Diagnostic and Statistical Manual of Mental Disorders, Fifth Edition; fNIRS: functional near-infrared spectroscopy; TMS: transcranial magnetic stimulation.

The study procedure is outlined in Figure 1. Prior to starting the study, participant and injury characteristics and questionnaires will be completed. Demographic information will be collected including age, sex, education level, and employment status. The participant characteristics collected will include headache history, concussion history, past medical history, medication use, and family medical history. Finally, injury characteristics will include length of loss of consciousness, length of posttraumatic amnesia, initial Glasgow Coma Scale, and imaging findings. The participants will be randomized to either active or sham rTMS. Once randomized, the participants will complete questionnaires and fNIRS. They will then complete 4 weeks (20 sessions) of treatment. Questionnaires and fNIRS will be repeated immediately following treatment and then at 1 month and 3 months post treatment.

### Blinding and Randomization

The participants will be randomized to either active or sham rTMS treatment by a research assistant external to the study using sequentially numbered opaque concealed envelopes. All individuals involved in the study except the research assistant administering the rTMS will be blinded to the treatment protocol, and allocation will be concealed. Following study completion, the participants and study personnel will be unblinded. Once recruitment is completed, if rTMS treatment proves beneficial after a full statistical analysis, the participants in the sham groups will be offered active rTMS treatment.

### TMS Protocol

The participants will engage in a 4-week treatment protocol (20 treatments). This was chosen as it reflects prior depression, PPCS, and migraine protocol durations [25-27]. A standardized atlas brain with Montreal Neurologic Institute coordinates will be used for navigation. rTMS treatment will be to the left DLPFC and will be located through Montreal Neurologic Institute coordinates (-50, 30, 36) [28]. The resting motor threshold will be determined using electromyography electrodes attached to the right abductor digiti minimi muscle and a TMS stimulation coil placed over the left motor cortex, as previously described by Stilling et al [11]. The intensity of the rTMS will be 100%-120% of resting motor threshold amplitude, with a frequency of 10 Hz, 10 trains of 60 pulses per train (total of 600 pulses) and intertrain interval of 45 seconds. In the sham condition, a sham coil will be applied to the scalp after the resting motor threshold is determined. Recent studies exploring rTMS implementing a 100%-120% resting motor threshold amplitude as a treatment for PPCS have been successful for patient retainment and treatment success [25,27,29,30]. Our group has previously used an intensity of 70% resting motor threshold in a similar patient population [11]. This protocol will use 120% resting motor threshold to attempt to achieve a greater neurophysiological effect [31]. Furthermore, previous studies treating posttraumatic headache and migraine show benefit using a frequency of 10 Hz, 600 pulses, and intertrain interval of 45 seconds, suggesting this protocol would also be beneficial in PPCS [32-35]. The sham coil will produce sounds and vibration similar to the real rTMS coil but will not produce any effective stimulation. This blinding method has been demonstrated to be effective in previous sham TMS studies [36].

### Functional Near-Infrared Spectroscopy

**Figure 2:**
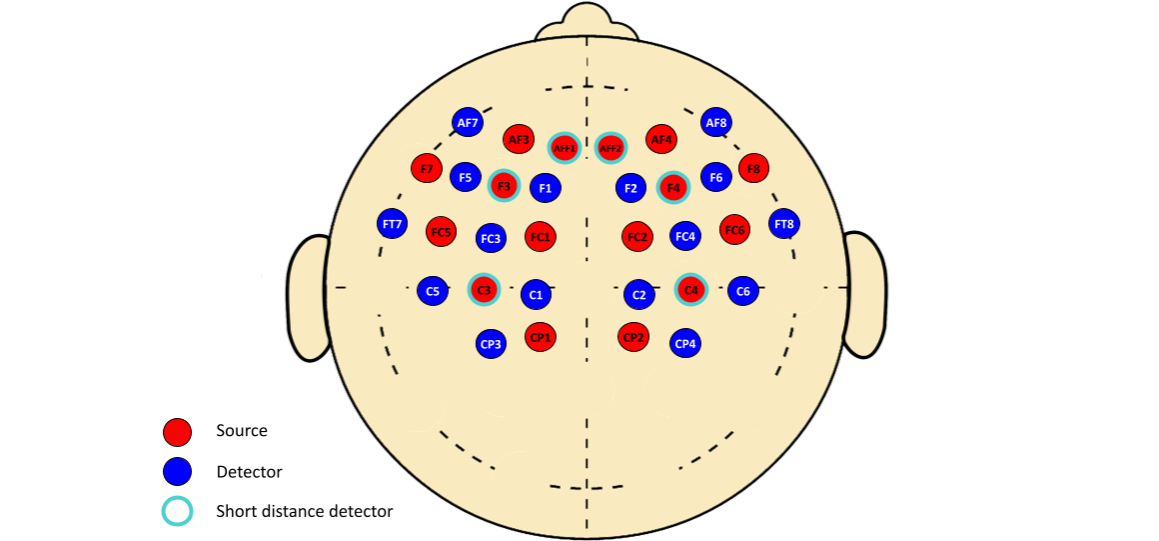
Optode configuration of functional near-infrared spectroscopy headcap based on 10-10 and 10-5 electroencephalogram coordinate system. AF: anterior frontal; AFF: anterior prefrontal; C: central; CP: central parietal; F: frontal; FC: fronto-central.

fNIRS will be completed before treatment, within 1 week following the 4-week treatment, and then at 1 month and 3 months post treatment. The TechEn (TechEn, Inc) system and a headcap with transmit and receive fibers positioned over the parietal and frontal lobes will be used (Figure 2). Data will be collected for 5 minutes at rest and then during a finger tapping exercise and working memory tasks. The resting task will consist of the participant sitting and resting while fixating on a small white cross for 5 minutes [37,38]. Finger tapping will be used as a simple, repeatable motor activation task, which has prominently shown a motor response [18,19]. The finger tapping exercise will consist of the participant tapping their right forefinger to their thumb at a frequency of 1 Hz, while being prompted on screen by “Tap” or a white cross indicating rest. The participant will alternate between tapping for 10 seconds and resting for 15 seconds, which will be repeated 10 times. Finally, the working memory task will consist of a 2-back variation of the n-back exercise [39], where a series of letters will appear on screen in 2-second intervals, and the participant will be asked to match each new letter to the letter presented 2 trials back. If the letters match, the participant is asked to press the right arrow key indicating correct, or, if the letters do not match, they are asked to press the left arrow key indicating incorrect. The participant will alternate between 20 presentations of letters followed by 20 seconds of rest, which will be repeated 3 times. Data on caffeine and other substance consumption from the last 8 hours before fNIRS assessment will also be collected. This protocol was adapted from the protocol described by Hocke et al [18].

### Rivermead Postconcussion Symptom Questionnaire

The RPQ is the primary outcome measure for this study. The RPQ assesses the severity of 16 commonly experienced PPCS symptoms. The participants are instructed to rate the extent to which they have suffered from each of the listed symptoms in the past 24 hours, as compared to preinjury levels, using a scale of 0 (“not experienced at all”) to 4 (“a severe problem”). It is advised to use this assessment as 2 separate scales (RPQ-13 and RPQ-3). Using these subscales, the instrument has good test-retest reliability and external construct validity [40]. This questionnaire probes the separate cognitive, emotional, and somatic components of PPCS.

### Exploratory Outcome Measures

Multiple other questionnaires will be administered as exploratory outcomes. The questionnaires administered will be described in the following section.

#### Quality of Life After Brain Injury Questionnaire

Quality of life will be measured by the quality of life after brain injury (QOLIBRI) questionnaire assessment. The QOLIBRI questionnaire is a health-related quality of life instrument developed specifically for participants who have experienced a traumatic brain injury. It has 6 subscales with a total of 37 items and is scored from 0 to 100 (with 100 being the best possible quality of life). This tool has been validated in the traumatic brain injury literature and is shown to have adequate (>0.5) to excellent (>0.7) test-retest reliability [41].

#### Headache Impact Test

The headache impact test-6 (HIT-6) is a 6-item tool used as a global measure of headache impact. The HIT-6 addresses the 6 categories of headache impact including social functioning, role functioning, cognitive functioning, vitality, psychological distress, and severity of headache pain. Each question is scored on a 5-point scale. The total score can range from 36 to 78, with higher total score indicating greater impact [42]. The HIT-6 has been validated in participants with episodic and chronic migraine [43]. Based on a chronic tension type headache study, a minimal clinically important change has been reported as a decrease of at least 8 points on the HIT-6 [44].

#### Patient Health Questionnaire-9

The patient health questionnaire-9 (PHQ-9) is a 9-item tool used to assess the presence and severity of depressive symptoms. In the PHQ-9, a score below 4 is classified as minimal, 5 to 9 is mild, 10 to 14 is moderate, 15 to 19 is moderate-to-severe, and anything higher than 20 is severe [45]. The PHQ-9 has been validated in participants with mild to severe traumatic brain injury with a screening cut-off score of greater than 12 for major depressive disorder [46]. The test-retest reliability is excellent within 7 days of the initial assessment. The minimal clinically important difference for the PHQ-9 is 5 points [47].

#### Generalized Anxiety Disorder Scale

The generalized anxiety disorder (GAD-7) scale is a 7-item tool where, like the PHQ-9, each item is rated on frequency over a 2-week period based on a 0 to 3 scale [48]. An analysis confirmed screens for depression (PHQ-9) and anxiety (GAD-7) to be distinct, and it is suggested that the 2 highly comorbid disorders be assessed independently [49].

#### Saint Louis University Mental Status Exam

The Saint Louis University mental status exam will be used to screen the participants for mild cognitive impairment. The Saint Louis University mental status exam is an 11-item scale with questions that correspond to attention, recall (immediate and delayed with interference), orientation, number calculation, memory, registration, visual spatial information, and executive function [50].

#### Screening for Somatoform Symptoms Conversion Disorder Subscale

Somatoform symptoms will be monitored through the screening for somatoform symptoms conversion disorder subscale. This screening lists 14 symptoms associated with somatization disorder. Each symptom is rated on degree of impairment experienced in the past 7 days on a 0 to 4 scale, with 0 indicating “not at all” and 4 indicating “very severe” [51].

#### Posttraumatic Stress Disorder Checklist for DSM-5

The posttraumatic stress disorder (PTSD) checklist for Diagnostic and Statistical Manual of Mental Disorders, Fifth Edition (DSM-5; PCL-5) is a screening tool used to assess PTSD. The PLC-5 is a 20-item self-report measure with each question scaled from 0 to 4, with the total score ranging from 0 to 80. A score greater than 53 may indicate more severe PTSD symptoms [52].

### Analysis

#### Preprocessing fNIRS Data

All preprocessing data will be done using MATLAB (The MathWorks Inc). Raw intensity data will first be converted to optical density using Homer3 (hmrR_Intensity2OD). Optical density will then be assessed for signal quality over several steps. First, channels that display low signal (below 90 dB) or have an oversaturated signal (above 140 dB) will be discarded. Channels that have a signal to noise ratio less than 1 will also be discarded, as previously described by Hocke et al [18]. Next, a spline motion correction step will be applied to correct for motion artifacts and baseline shifts using Homer3 (hmrR_MotionCorrectSpline). Short distance detectors located approximately 8 mm from select sources will be used to remove the influence of extracerebral signal obtained from superficial layers [53,54]. A bandpass filter will be applied to the data with a low pass filter of 0.01 Hz and a high pass filter of 2 Hz (hmrR_BandpassFilt). Optical density data will then be converted to delta concentration of oxyhemoglobin and deoxyhemoglobin using the modified Beer-Lambert law with age-dependent differential path length factors. Finally, the concentration data will be down sampled to 5 Hz.

#### Postprocessing fNIRS Data

Preprocessed data between 4 major brain regions (ie, left DLPFC, left motor cortex, right DLPFC, and right motor cortex) will be assessed using a coherence analysis at 0.04 to 0.1 Hz. Coherence will be calculated for all channels over the 4 regions. Coherence is defined as an estimate of a linear-time variant relationship between 2 signals at a particular frequency domain [19]. The coherence value used will be the maximum coherence between each combination of regions. The interregional coherence values will be quantified between the right and left DLPFC, the left and right motor cortex, the left DLPC and left motor cortex, and finally between the right DLPFC and right motor cortex [18].

#### Statistical Analysis

The effect size of the RPQ score extrapolated from a similar study (*d*=0.77) [10] provided the basis for a power calculation performed with an alpha of .05, and 80% power suggested that 44 participants should be included in this study (22 per group). Anticipating a 20% attrition [10,22,55], 56 participants will be recruited for this study.

Descriptive statistics will be used to analyze baseline characteristics. A 2-way (treatment × time) mixed ANOVA will be used to assess whether any changes in clinical measurements and outcome parameters were the result of the interaction of type of treatment (real vs sham) and time. fNIRS data will also be analyzed using a 2-way mixed ANOVA where interregional coherence will act as the dependent variable, sham vs real TMS treatment will act as the between-subject variable, and brain region and assessment (ie, pre-TMS, post-TMS, 1-month follow-up, and 3-month follow-up) will act as the within subject variables. A *P* value of ≤.05 will be considered significant. A post hoc analysis will be used for exploratory measures. Simple effects testing using a Bonferroni correction will be performed when a significant group by time interaction is detected.

fNIRS data will be assessed to determine the location and magnitude of task-evoked changes in oxyhemoglobin, deoxyhemoglobin, and total hemoglobin at the left DLPC for each participant. Coherence of hemodynamic frequencies between major brain regions will also be quantified to observe brain connectivity during tasks as previous works have done [18,19]. Our group has previously attempted to decrease bias by restricting change in medication and other treatments throughout the trial [11]; however, it is unknown how some medications may influence rTMS and fNIRS data. Full patient characteristics, injury characteristics, and medication profile will be collected to better understand this bias.

## Results

Participant recruitment for the study started on March 25, 2021. To date, 5 participants are enrolled, and 3 have completed the rTMS protocol as of May 6, 2021. The study protocol will be completed in May 2022. The results are expected in June 2022 with submitted manuscript in August 2022.

## Discussion

The proposed project will substantially contribute to the growing body of literature exploring rTMS as a safe and noninvasive treatment for participants with PPCS. The mechanism by which rTMS influences brain function and subsequently alters participants’ symptoms and function is unknown. fNIRS analysis will provide insight into the neuropathophysiological changes that can occur following rTMS treatment in participants with PPCS. Several studies have explored fNIRS and TMS in the clinical setting outside of PPCS [56] including depression [57], panic disorder [58-60], tinnitus [61], phobias [62], bulimia nervosa [63], and stroke [64-66]. Our group has already shown through a 2-patient case study that fNIRS might be useful as a sensitive tool to predict response to rTMS in participants with postconcussion syndrome. This study will help grow the understanding of rTMS as a treatment for PPCS and provide additional support for the use of fNIRS as a tool to understand the effects of neuromodulation on participants with PPCS.

## Abbreviations

DLPFC: dorsolateral prefrontal cortex
DSM-5: Diagnostic and Statistical Manual of Mental Disorders, Fifth Edition
fNIRS: functional near-infrared spectroscopy
GAD-7: generalized anxiety disorder scale
HIT: headache impact test
ICD: International Classification of Diseases
PCL-5: posttraumatic stress disorder checklist for DSM-5
PHQ-9: patient health questionnaire
PPCS: persistent postconcussion symptom
PTSD: posttraumatic stress disorder
QOLIBRI: quality of life after brain injury questionnaire
RPQ: Rivermead postconcussion symptom questionnaire
rTMS: repetitive transcranial magnetic stimulation
TMS: transcranial magnetic stimulation
